# Modulatory Effects of Bioactive Phytoconstituents on the Amplitude and Gating Properties of Membrane Ion Channels

**DOI:** 10.3390/molecules31081360

**Published:** 2026-04-21

**Authors:** Sheng-Nan Wu, Guglielmina Froldi, Ya-Jean Wang, Rasa Liutkevičienė

**Affiliations:** 1 Department of Medical Research, An Nan Hospital, China Medical University, No. 66, Section 2, Changhe Road, An Nan District, Tainan 70965, Taiwan; 2 Department of Pharmaceutical and Pharmacological Sciences, University of Padova, Largo E, Meneghetti 2, 35131 Padova, Italy; g.froldi@unipd.it; 3 Department of Senior Service Industry Management, Minghsin University of Science and Technology, 1 Xinxing Road, Xinxfen Township, Hsinchu 304001, Taiwan; yawang@must.edu.tw; 4 Laboratory of Ophthalmology, Institute of Neuroscience, Lithuanian University of Health Sciences, 50106 Kaunas, Lithuania; rasa.liutkeviciene@lsmuni.lt

**Keywords:** phytoconstituents, voltage-gated channels, ionic current, ion channel, natural products, gating kinetics

## Abstract

This review provides a comprehensive overview of the modulatory actions of plant-derived constituents on membrane ion channels in various cell types. Among their diverse bioactivities, ion channel regulation—governing membrane excitability, signal transduction, and cellular homeostasis—has emerged as a critical mechanistic basis for their pharmacological effects. Twenty-four representative phytoconstituents are discussed and classified into five major categories based on their structural features: alkaloids, terpenoids, lignans and acetogenins, polyphenols, and other aromatic and conjugated compounds. Across these categories, the reviewed compounds exhibit distinct and often highly specific effects on the amplitude and gating kinetics of multiple ionic currents, including voltage-gated Na^+^ currents (*I*_Na_), delayed-rectifier K^+^ currents (*I*_K(DR)_), M-type K^+^ currents (*I*_K(M)_), hyperpolarization-activated cation currents (*I*_h_), erg-mediated K^+^ currents (*I*_K(erg)_), inwardly rectifying K^+^ currents, and Ca^2+^-activated K^+^ currents (*I*_K(Ca)_). Alkaloids predominantly suppress voltage-gated K^+^ currents, with notable exceptions such as aconitine, which alters the properties of both *I*_Na_ and *I*_K(DR)_, thereby contributing to its proarrhythmic toxicity. Terpenoids, including cannabidiol, croton diterpenoids, lutein, thymol, and triptolide, exert multifaceted effects on *I*_K(M)_, *I*_h_, inwardly rectifying K^+^ currents, and Ca^2+^-activated K^+^ channels. Lignans and acetogenins, such as gomisin A, honokiol, sesamin, and squamocin, primarily modulate *I*_Na_, *I*_h_, and *I*_K(Ca)_, with several compounds demonstrating strong links between ion-channel modulation and anti-neoplastic or neuroprotective actions. Polyphenolic compounds, including curcumin, eugenol, resveratrol, gastrodigenin, gastrodin, and pterostilbene, display diverse ion-channel targeting profiles, influencing multiple Na^+^ and K^+^ channel subtypes. Other aromatic or conjugated compounds, such as isoplumbagin, plumbagin, and verteporfin, regulate *I*_K(erg)_ and *I*_K(Ca)_, potentially contributing to both therapeutic efficacy and adverse effects. Collectively, the compound-specific modulation of current amplitude and gating kinetics offers valuable mechanistic insight into the pharmacological and toxicological significance of plant-derived natural products, highlighting the functional role of ion channel evaluation in guiding their therapeutic development and ensuring safety assessment.

## 1. Introduction

**Figure 1:**
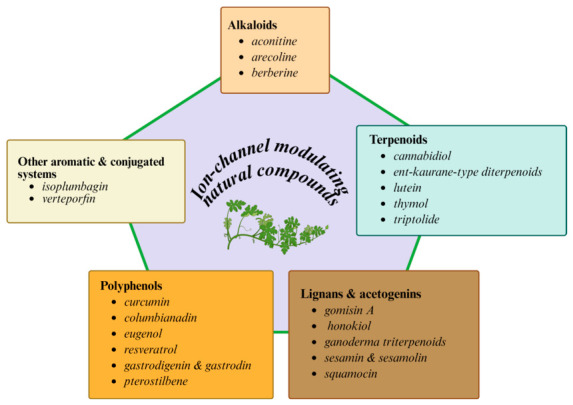
Graphic representation showing the five major categories of phytoconstituents and their potential modulation of ion channels. The five categories are alkaloids (pink), terpenoids (light blue), lignans and acetogenins (brown), polyphenols (orange), and other aromatic and conjugated systems (light yellow). Within each box, the phytochemicals discussed in this article are listed individually.

Plant-derived natural products, often referred to as phytochemicals, are secondary metabolites that plants produce to protect themselves from environmental stress, pests, and diseases. These compounds provide humans with a vast repertoire of bioactive properties that may contribute to the prevention or treatment of a wide range of diseases and disorders [1,2,3,4]. Among these bioactive properties, their regulatory effects on ion channels in the cell membranes of different cell types have long been an important subject of academic research. This review paper examines 24 phytoconstituents derived from plant natural products. Based on their major structural features, these compounds are broadly classified into five main categories: alkaloids, terpenoids, lignans and acetogenins, polyphenols, and other aromatic and conjugated systems (Figure 1). We present a comprehensive overview of the currently recognized modulatory effects of these compounds on membrane ion channels across a broad range of mammalian cell types.

The GH_3_ cells employed in this study are immortalized rat pituitary somatolactotrophs derived from a pituitary adenoma and are considered excitatory endocrine cells [5,6,7]. In pituitary cells, somatolactotrophs are classified as excitatory endocrine cells, whereas other types of pituitary cells are considered inhibitory endocrine cells. Excitatory endocrine cells are characterized by a high intrinsic level of excitability under basal conditions and are typically subject to suppression by inhibitory neurotransmitters, such as dopamine. In contrast, inhibitory endocrine cells exhibit low basal excitability and require stimulation by excitatory hormones to become activated [8]. It is important to note that the pituitary gland contains a wide variety of cell types, including corticotrophs, thyrotrophs, gonadotrophs, melanotrophs, somatotrophs, and lactotrophs [6]. Therefore, isolating and obtaining healthy pituitary cells remains technically challenging, and performing electrophysiological studies on these primary cells is even more difficult. Successfully isolating and efficiently obtaining immortalized cell lines derived from the human pituitary gland, and performing electrophysiological characterization of these cells, will constitute an important task for future research [6,8].

Alternatively, regarding treatment, pituitary tumors are primarily treated through surgical resection, most commonly performed using a transsphenoidal approach, as well as stereotactic radiosurgery techniques such as Gamma Knife. In cases where residual tumor tissue remains after surgery, various pharmacological therapies are employed to reduce the risk of recurrence or further growth [9,10]. Notably, voltage-dependent ion channels have emerged as promising potential therapeutic targets for future treatment strategies [6,11,12]. In addition to GH_3_ cells, this review also highlights the utility of other cell types, including insulinoma cells, glioma cells, Leydig tumor cells, and Rolf B1.T olfactory neurons.

## 2. Alkaloids

### 2.1. Aconitine

Aconitine is an intensely poisonous alkaloid derived from plants of the genus *Aconitum* (Ranunculaceae) [13,14]. This compound is a proarrhythmic agent known to open tetrodotoxin-sensitive Na^+^ channels in electrically excitable cells [15]. Aconitine-containing herbal extracts have also been reported to exert inhibitory effects on the proliferation of various neoplastic cells [6,8]. Studies have reported that this compound suppresses the delayed-rectifier K^+^ current (*I*_K(DR)_) in neurons, cardiomyocytes, and immune cells, leading to current inactivation upon membrane depolarization [16,17,18,19]. Recent findings indicate that aconitine can induce both early and delayed afterdepolarizations in neonatal cardiac cells and can trigger ventricular polymorphic tachycardia in rats [17,19]. These findings underscore the importance of understanding the electrophysiological impact of aconitine and similar compounds, considering their potential therapeutic applications and associated toxicities. Prior to evaluating the therapeutic potential of *Aconitum* extracts, such as their antitumor effects, it is essential to thoroughly assess the possible toxicities associated with these reagents.

### 2.2. Arecoline

Areca nut, derived from *Areca catechu* (family Arecaceae), has been widely used in China for its medical properties and psychoactive stimulant effects [20]. However, it is also recognized as a major risk for the development of oral cancer. Arecoline, a principal alkaloid isolated from the areca nut (chemically defined as 1,2,5,6-tetrahydro-1-methyl-3-pyridinecarboxylic acid methyl ester), acts as a non-selective agonist of muscarinic receptors. Extensive studies have demonstrated that arecoline exhibits carcinogenic, cytotoxic, and immunotoxic effects [21,22].

Arecoline has been reported to exert neuroprotective effects in the context of Alzheimer’s disease [23]. It also promotes the production of connective tissue growth factor in human buccal mucosal fibroblasts and induces apoptosis in HaCaT keratinocytes [21,22]. Previous studies have shown that, in U373 and U87MG glioma cells, arecoline inhibits intermediate-conductance Ca^2+^-activated K^+^ channels (K_Ca_3.1; encoded by *KCNN4*) in a concentration-, voltage- and state-dependent fashion [24]. The dissociation constant for this inhibitory action was estimated to be 11.2 μM [24]. The resulting membrane depolarization induced by arecoline is therefore primarily attributable to its suppression of K_Ca_3.1 channel activity in these cells. Notably, this effect appears to occur independently of muscarinic receptor activation [24]. Compounds such as arecoline that target K_Ca_3.1 channels may thus be further developed as promising adjunctive agents to enhance the efficacy of cytotoxic therapies for malignant gliomas and other neoplastic diseases [25].

### 2.3. Berberine

Berberine is an isoquinoline alkaloid present in a variety of medicinal plants, particularly those belonging to the genera *Berberis* (Berberudaceae) and *Coptis* (Ranunculaceae). Extracts from these plants have long been used in traditional medicine for the treatment of conditions such as jaundice, dysentery, hypertension, and other ailments [26]. In addition, berberine itself has been shown to exert anti-proliferative effects on human teratocarcinoma and hepatoma cells [27].

An earlier study demonstrated that, in human myeloma cells, berberine directly exerts differential inhibitory effects on delayed-rectifier K^+^ current (*I*_K(DR)_) and Ca^2+^-activated K^+^ current (*I*_K(Ca)_) in a concentration-dependent manner [28]. Consistent with these electrophysiological findings, berberine markedly suppresses cellular proliferation. The close correspondence between the potency (IC_50_ values) for K^+^ current inhibition and for the reduction in cell proliferation suggests that block of *I*_K(DR)_ and *I*_K(Ca)_ may represent a key mechanism underlying its antineoplastic activity [28]. Beyond its effects on ion channels, berberine has also been reported to enhance mitochondrial function and to upregulate pro-apoptotic proteins, including cytochrome c, Bax, and caspase. Collectively, these findings indicate that berberine is a biologically active compound with considerable therapeutic potential for the treatment of a range of diseases, including glioma and Alzheimer’s disease [29,30,31].

## 3. Terpenoids

### 3.1. Cannabidiol

Cannabidiol, which is considered a terpenophenol, is a non-psychoactive cannabinoid derived from the *Cannabis* plant (Cannabaceae), known for its potential therapeutic effects. It is among over 100 cannabinoids present in the plant and has been shown to be effective in treating various medical conditions, such as epilepsy, bipolar disorder, inflammation, and cancer [32]. Recent studies have demonstrated that cannabidiol influences the activity in the hypothalamic–pituitary–adrenal axis and modulates several ionic currents in excitable cells, including voltage-gated Na^+^ currents (*I*_Na_), M-type K^+^ currents (*I*_K(M)_), and hyperpolarization-activated cation currents (*I*_h_) [33,34,35].

Cannabidiol exposure was noted to result in a concentration-dependent suppression of *I*_K(M)_ in pituitary GH_3_ lactotrophs, with an IC_50_ of 3.6 μM [35]. This compound also results in the suppression of *I*_h_ in the same cell type with an IC_50_ of 3.3 μM [35]. These findings suggest that the responsiveness of these ionic currents is complex and influenced by various factors, including the resting membrane potential, cannabidiol concentration, patterns of action potential firing, or combinations of these variables [35].

### 3.2. Ent-Kaurane-Type Diterpenoids

The genus *Croton* (Euphorbiaceae) includes about 1300 species that are widely distributed throughout tropical regions. *C. tonkinensis* Gagnep., known as *Kho sam cho la* in Vietnamese, is a tropical shrub native to northern Vietnam and has been used commonly in Vietnam to treat different disorders [36]. *C. tonkinensis* is a rich source of diterpenoids [36], and its extracts were reported to exert anti-inflammatory and cancer chemopreventive activities [36,37].

Studies have shown that the fractions isolated from *C. tonkinensis*—specifically croton-01 (*ent*-18-acetoxy-7α-hydroxykaur-16-en-15-one), croton-02 (*ent*-7α,14β-dihydroxykaur-16-en-15-one), and croton-03 (*ent*-1β-acetoxy-7α,14β-dihydroxykaur-16-en-15-one)—are capable of modulating ionic currents in microglial SM826 cells, including the *I*_K(DR)_ (encoded by K_V_1.3) and the inwardly rectifying K^+^ current (encoded by Kir2.1) [38]. The notable findings showed that in these microglial cells, croton-03 differentially suppresses K_V_1.3- and Kir2.1-encoded currents in a concentration-, time-, and state-dependent manner. Voltage-dependent blocking by croton-03 of K_V_1.3-encoded current with K_d_ (apparent affinity constant) and δ (fractional electrical distance) values of 5.17 μM and 0.091, respectively, was also estimated [39]. Therefore, the presence of these *ent*-kaurane diterpenoids is likely to disrupt cellular function by modulating the activity of these ionic currents [37,39].

Another report further demonstrated that the presence of croton-01, croton-02, and croton-03 produces concentration-dependent inhibition of *I*_h_ in pituitary tumor (GH_3_) cells with effective IC_50_ values of 2.89, 6.25, and 2.84 μM, respectively [40]. Cell exposure to croton-03 was also noted to decrease the voltage-dependent hysteresis of this current in response to long-lasting isosceles triangular ramp pulse [40]. Therefore, croton derivatives and other structurally related *ent*-kaurane-type diterpenoids could represent intriguing compounds that use the open/activated state of the HCN channels as a substrate.

### 3.3. Lutein

Lutein (a xanthophyll carotenoid; β,ε-carotene-3,3′-diol), derived from a (6R)-β,ε-carotene backbone, is one of the few xanthophylls that is found not only in fruits and vegetables but also at high concentrations in the macula of the human retina, where it is believed to function as a yellow light filter [41]. Previous work has shown that, in pituitary GH_3_ cells, exposure to lutein suppresses the hyperpolarization-activated cation current (*I*_h_) in a concentration-, state-, voltage-, and hysteresis-dependent manner [42]. In addition, lutein induces a hyperpolarizing shift in the steady-state activation curve of this current.

**Figure 2:**
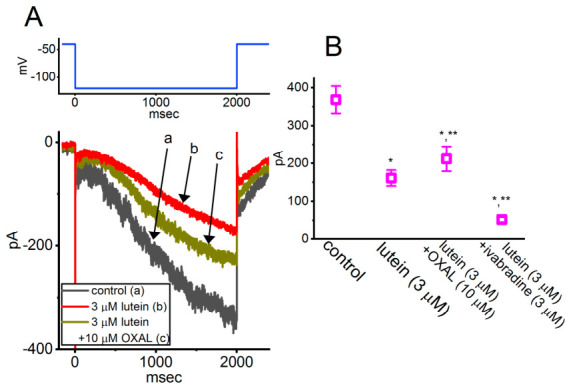
Comparative effects of lutein, lutein plus oxaliplatin (OXAL), and lutein plus ivabradine on the amplitude of hyperpolarization-activated cation current (*I*_h_) recorded from pituitary GH_3_ cells. Whole-cell current recordings were obtained from cells superfused with Ca^2+^-free Tyrode’s solution containing 1 μM tetrodotoxin, while the recording pipette was filled with a K^+^-rich internal solution. (**A**) Representative superimposed *I*_h_ traces recorded under control conditions (a, black), in the presence of 3 μM lutein alone (b, red), and during co-application of 3 μM lutein with 10 μM oxaliplatin (OXAL) (c, brown). The upper part shows the voltage protocol (blue) applied to the cell. Notably, *I*_h_ displays slow activation during sustained hyperpolarization. (**B**) Summary scatter plot illustrating the effects of lutein, lutein plus OXAL, and lutein plus ivabradine on *I*_h_ amplitude (mean ± SEM; *n* = 7 for each point). Current amplitudes were measured at the end of a 2-s hyperpolarizing step from −40 to −120 mV. For combination treatments, lutein was applied prior to the subsequent addition of OXAL or ivabradine. * *p* < 0.05 versus control; ** *p* < 0.05 compared with the lutein-alone (3 μM) group. This figure is adapted from Ref. [42] and is distributed under the Creative Commons Attribution (CC BY) license.

**Figure 3:**
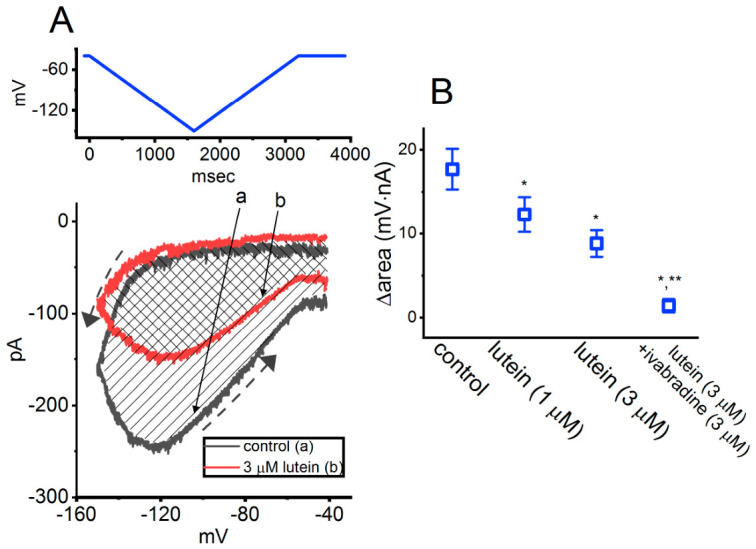
Modulation by lutein of voltage-dependent hysteresis strength in *I*_h_ recorded from GH_3_ cells. (**A**) Representative current traces illustrating the relationship between forward (descending) or reverse (ascending) *I*_h_ and membrane potential, evoked by an inverted double (isosceles-triangular) ramp pulse (protocol shown at the top, blue) in the absence (a, black) and presence (b, red) of 3 μM lutein. The dashed black arrows along the current trajectory under control conditions (i.e., in the absence of lutein) indicate an anti-clockwise direction of *I*_h_, reflecting time-dependent changes during activation measured in the absence or presence of lutein. Notably, *I*_h_ exhibits inward rectification along with pronounced voltage-dependent hysteresis. (**B**) Summary data showing the effects of lutein (1 or 3 μM) and lutein (3 μM) in combination with ivabradine (3 μM) on the Δarea of voltage-dependent hysteresis. The Δarea corresponds to the shaded region enclosed by the *I*_h_ trajectories during the downsloping and upsloping limbs of the triangular ramp pulse (mean ± SEM; n = 7 for each point). The results demonstrate the presence of ramp pulse induced hysteresis in *I*_h_, and lutein reduced the Δarea in concentration-dependent manner. * *p* < 0.05 compared with control; ** *p* < 0.05 compared with the lutein-alone (3 μM) group. This figure is adapted from Ref. [42] and is distributed under the Creative Commons Attribution (CC BY) license.

Furthermore, in the continued presence of 3 μM lutein, the subsequent application of oxaliplatin or ivabradine was able to modulate the lutein-induced inhibition of *I*_h_ in GH_3_ cells, as illustrated in Figure 2. Oxaliplatin and ivabradine have been reported to enhance and suppress *I*_h_, respectively [43,44]. Cyclic nucleotide-gated (CNG) channels (e.g., CNGA2) have previously been shown to exhibit pronounced hysteresis in their ion currents [45]. As demonstrated in Figure 3, the results further reveal that the magnitude of *I*_h_ elicited by a double-ramp pulse protocol can influence the patterns of burst firing in various excitable cells, including central neurons [42,46].

During these current measurements, the tested cell was held at −40 mV and a long-lasting inverted double ramp pulse was applied [43]. This ramp protocol consists of a downsloping (forward) phase from −40 to −150 mV, followed by an upsloping (backward) return to −40 mV, with a total duration of 3.2 s (corresponding to a ramp speed of ±69 mV/s), as illustrated in the upper part of Figure 3A. As shown in Figure 3, voltage-dependent hysteresis of *I*_h_—defined as the relationship between *I*_h_ and membrane potential during the forward and backward limbs—was evident upon activation by this triangular double ramp pulse [47].

Notably, the *I*_h_ amplitude evoked during the downsloping phase of the inverted triangular ramp was smaller (in absolute value) than that elicited during the upsloping phase, as indicated by the dashed arrows along the current trajectory (Figure 3A). For example, under control conditions (i.e., in the absence of lutein), the *I*_h_ amplitude measured at −120 mV differed significantly (*p* < 0.05) between the two phases, with values of 48 ± 8 pA during the downsloping limb and 249 ± 11 pA during the upsloping limb.

Upon exposure to lutein (3 μM), *I*_h_ recorded during the downsloping limb showed a small reduction compared to that observed during the upsloping limb of the triangular ramp pulse. Consequently, the extent of lutein-induced current inhibition of *I*_h_ differed significantly between the forward and reverse phases of the ramp protocol. As further quantified in Figure 3B, the degree of *I*_h_ hysteresis in GH_3_ cells, in the absence or presence of lutein, was assessed by measuring the ∆area under the curve (i.e., the shaded region in A) [42].

Specifically, in addition to reducing *I*_h_ magnitude, application of 3 μM lutein significantly decreased the Δarea associated with the inverted triangular ramp pulse, from 17.7 ± 2.4 to 8.8 ± 1.6 mV·nA (n = 7, *p* < 0.05). Following washout of lutein, the hysteretic area recovered to 17.2 ± 2.3 mV·nA. Moreover, during continued exposure to 3 μM lutein, subsequent addition of 3 μM ivabradine produced a further significant reduction in the ∆area of voltage-dependent hysteresis.

**Figure 4:**
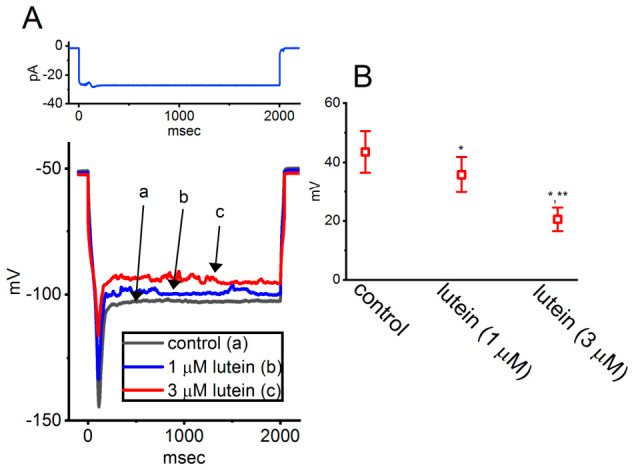
Effect of lutein on sag potential in GH_3_ cells. Current-clamp recordings were performed in cells bathed in normal Tyrode’s solution containing 1.8 mM CaCl_2_ and 1 μM tetrodotoxin. A prolonged hyperpolarizing current step (2 s duration) was applied to evoke sag potential. (**A**) Representative membrane potential traces obtained under control conditions (a, black) and during exposure to 1 μM lutein (b, blue) or 3 μM lutein (c, red). The upper part shows the corresponding hyperpolarizing current stimulus applied to the recorded cell. (**B**) Summary scatter plot illustrating the effect of lutein on sag potential amplitude (mean ± SEM; n = 7 for each point). Under current-clamp conditions, sag amplitude was defined as the difference between the initial peak hyperpolarization and the steady-state level at the end of the current pulse, measured in the absence or presence of lutein. * *p* < 0.05 versus control; ** *p* < 0.05 compared with the lutein-alone (1 μM) group. This figure is adapted from Ref. [42] and is distributed under the Creative Commons Attribution (CC BY) license.

To further investigate the effects of lutein on sag potential in GH_3_ cells, the researchers employed current-clamp recordings [42]. Sag potential, elicited by hyperpolarizing current stimuli, is known to be closely associated with the presence of *I*_h_ in various types of excitable cells [47,48,49,50]. As illustrated in Figure 4, applying a 2-s hyperpolarizing current step of approximately pA induced a characteristic sag potential, defined as an initial rapid hyperpolarization followed by a gradual depolarizing “sag” in membrane potential during sustained current injection [49].

Exposure to ivabradine (3 μM), a known *I*_h_ inhibitor, significantly reduced the amplitude of sag potential. Similarly, treatment with lutein at concentrations of 1 or 3 μM markedly attenuated the sag potential evoked by the hyperpolarizing stimulus [42].

These findings suggest that, in addition to its established antioxidative and anti-inflammatory properties [51], lutein can modulate *I*_h_ by reducing its amplitude and altering its gating and hysteresis behavior. Consequently, lutein’s effects on ionic currents may contribute to changes in spontaneous action potential activity in electrically excitable cells, assuming similar effects occur in vivo [42].

### 3.4. Thymol

Thymol, a monoterpenoid phenol derived from thyme (*Thymus vulgaris*, Lamiaceae) essential oil, has been widely used as an antiseptic and antimicrobial agent [52,53,54,55]. Its aromatic properties make it a common ingredient in mouthwashes and dental preparations for treating oral infections [52]. Beyond oral care applications, thymol serves as a stabilizer in several therapeutic agents, including the anesthetic halothane, where accumulates in vaporizers during prolonged use. Notably, adverse effects following massive mouthwash ingestion have been attributed to thymol [56,57].

Previous studies have revealed that its biological activities may be partly mediated through modulation of ion channels. In particular, previous studies indicate that thymol influences Ca^2+^ signaling in pituitary GH_3_ cells by enhancing capacitative Ca^2+^ entry and depleting intracellular Ca^2+^ stores, involving both thapsigargin-sensitive and thapsigargin-insensitive pools [58]. Moreover, thymol was reported to stimulate *I*_K(Ca)_ in GH_3_ cells. These stimulatory effects on Ca^2+^ dynamics may contribute to the cellular mechanisms underlying the impact of thymol on neuroendocrine or endocrine functions.

### 3.5. Triptolide

Triptolide is a diterpene triepoxide derived from the traditional Chinese medicinal plant *Trypterygium wilfordii* Hook. F. (Celastraceae). It exhibits a wide range of biological activities, including antitumor, immunosuppressive, and antifertility effects [59]. In the central nervous system (CNS), triptolide has been shown to exert neuroprotective properties and to promote axon growth in dopaminergic neurons [60]. Additionally, it facilitates spinal cord repair in animal models of spinal cord injury by reducing astrogliosis and inflammation [61]. Triptolide has also been reported to inhibit the proliferation and invasion of malignant glioma cells [62] and to induce apoptosis in these cells [63]. Owing to its low molecular weight and high lipid solubility, triptolide can readily cross the blood–brain barrier, enabling it to exert significant effects on glial and glioma cells [60,64].

Previous studies showed that in human glioma cells (U373 cells), triptolide inhibits the inwardly rectifying K^+^ current in a concentration-dependent manner with an IC_50_ of 0.72 μM [64]. Triptolide suppresses the activity of the inwardly rectifying K^+^ channels in these cells with no change in single-channel conductance. The biophysical properties of the inwardly rectifying K^+^ currents in U373 cells resemble the Kir4.1-encoded current because of positive mRNA detection of KCNJ10 (Kir4.1) and high sensitivity to inhibition by BaCl_2_. KCNJ10, the gene that codes for Kir4.1, is recognized as a putative seizure susceptibility gene in mice and humans [64,65]. Therefore, the pharmacological actions of triptolide as described recently [59,60,61,62,63] could be partly, if not entirely, connected with its inhibition of the inwardly rectifying K^+^ channels in glial or glioma cells. However, it should be noted that mRNA expression for other Kir channels may also occur in U373 cells.

**Figure 5:**
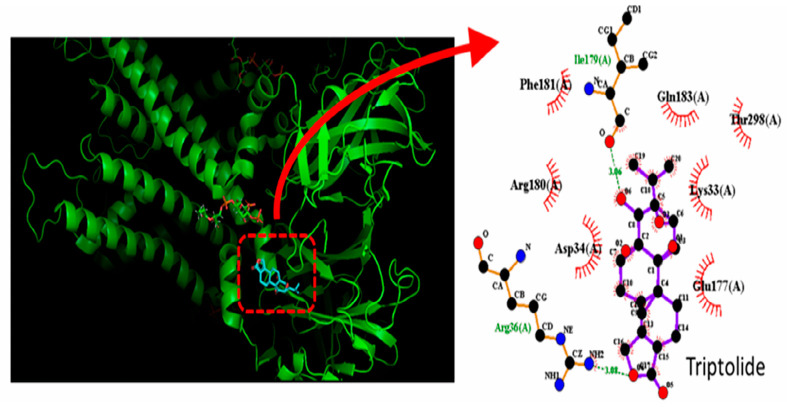
Docking interaction between the triptolide molecule and the KCNJ10 (or Kir4.1-encoded) channel. The left graph refers to the docking prediction between triptolide and the KCNJ10 channel. The protein structure of KCNJ10 was obtained from the Protein Data Bank (PDB ID: 8I5M), and the three-dimensional chemical structure of triptolide was retrieved from PubChem (Compound CID: 107985). On the left, the red dashed box highlights a snapshot of triptolide engaging hydrophobic interactions and forming hydrogen bonds with the channel, which is enlarged and illustrated on the right. The green dashed line indicates hydrogen bond formation, with bond lengths estimated at 3.06 and 3.08 Å. Of note, in this and the following figures showing predicted docking, the red arcs, with spokes pointing toward the triptolide molecule, denote hydrophobic contacts, and the corresponding graph in the right panel indicates an expanded display of red dashed box with a curve arrow in the left panel.

We conducted a detailed analysis of the atomic interaction between the KCNJ10 protein [66] and triptolide using PyRx 0.8 software, employing its embedded AutoDock (PyRx 0.8; AutoDock Vina 11.2) function. Figure 5 illustrates the predicted docking sites of the triptolide molecule. Notably, during the docking analysis with the KCNJ10 channel, triptolide was observed to form hydrogen bonds with residues Arg36(A) and Ile179(A), with bond lengths of 3.08 and 3.06 Å, respectively. Moreover, triptolide exhibited hydrophobic interactions with several residues, including Lys33(A), Asp34(A), Glu177(A), Arg180(A), Phe181(A), Gln183(A), and Thr198(A). These findings suggest a strong binding affinity between triptolide and the amino acid residues of the KCNJ10 channel, estimated at −7.8 kcal/mol. This interaction predominantly occurs within the intracellular domain of chain A in the KCNJ10 channel. This predicted interaction therefore raises the possibility that triptolide-mediated alterations in the magnitude and gating kinetics of the inwardly rectifying K^+^ currents may occur independently of its binding to muscarinic receptors [67].

## 4. Lignans and Acetogenins

### 4.1. Gomisin A

Gomisin A (also known as wuweizichu B, wǔwèizi chún yĭ) is a dibenzocyclooctadiene lignan isolated from the hexane fraction of the fruits of *Schisandra chinensis* (Schisandraceae) [68]. This dietary compound exhibits a broad spectrum of pharmacological activities, including anti-inflammatory, anti-oxidant, antihypertensive, neuroprotective, and antiproliferative effects [68]. Previous studies have demonstrated that gomisin A confers protective effects against hepatic and renal injury induced by carbon tetrachloride (CCl_4_), as well as against striatal toxicity caused by nitropropionic acid, primarily through modulation of the MAPK signal transduction pathway [69,70].

In addition, gomisin A suppresses the expression of key inflammatory mediators—including COX-2, iNOS, IL-6, TNF-α and NOּ—by inhibiting RIP2-mediated activation of the NF-κB activation in mouse macrophages [71]. It has also been reported to exert antioxidative effects during osteoblast differentiation and in vascular endothelial cells [68,72]. Furthermore, gomisin A can induce apoptotic responses in human colon carcinoma HCT-116 cells [73], highlighting its potential anticancer properties.

Pharmacokinetic studies have indicated that active lignan components of *S. chinensis* are detectable in vivo following intragastric administration in rats [67], supporting their bioavailability. Moreover, crude extracts of *S. chinensis* have been shown to reduce prolactin production in pituitary GH_3_ cells, suggesting therapeutical applications in the treatment of hyperprolactinemia and prolactin-secreting tumors [74].

A notable study reported that gomisin A exerts a suppressive effect on voltage-gated Na^+^ currents (*I*_Na_) in GH_3_ cells in a concentration-, time- and state-dependent manner. Exposure to gomisin A was found to accelerate the inactivation kinetics of *I*_Na_, with a particularly pronounced effect on the slow component of current inactivation [75]. The inhibitory action developed rapidly and was readily reversible upon washout. This effect was temporally associated with a marked increase in the inactivation rate of currents evoked by brief depolarizing pulses [75,76], while the activation kinetics of *I*_Na_ remained largely unaffected.

Further analysis revealed that, in GH_3_ cells, gomisin A differentially inhibits the transient or late components of *I*_Na_, with IC_50_ value of 6.2 or 0.73 μM, respectively [75], indicating a greater potency toward the late current component. The modulatory effects of gomisin A on both the amplitude and gating properties of *I*_Na_ are therefore thought to arise from its direct interaction with voltage-gated Na^+^ (Na_V_) channel or their accessory subunits [76].

**Figure 6:**
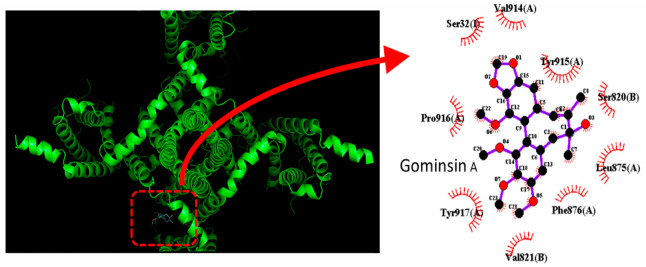
Docking analysis of Na_V_1.7 (SCN9A) and gomisin A. The protein structure of SCN9A was retrieved from the Protein Data Bank (PDB ID: 6N4Q), while the three-dimensional structure of gomisin A was obtained from PubChem (CID: 15608605). Molecular docking was performed using PyRx, and the optimal binding pose of gomisin A within SCN9A is highlighted by the red dashed box on the left panel.

Subsequently, molecular docking analysis was performed to evaluate the interaction between the SCN9A (Na_V_1.7) channel protein and gomisin A using PyRx 0.8 software. The predicted binding sites of gomisin A on the channel are depicted in Figure 6. The docking results indicate that gomisin A forms hydrophobic interactions with multiple amino acid residues, including Ser32(I), Ser820(B), Val821(B), Leu875(A), Phe876(A), Val914(A), Tyr915(A), Pro916(A), and Tyr917(A), which are primarily located within the membrane-embedded regions of the channel protein.

Given the experimentally observed inhibitory effect of gomisin A on *I*_Na_, together with the predicted binding interactions with the SCN9A protein, these findings suggest that gomisin A may directly modulate Na_V_1.7 channel activity. Consequently, this compound holds potential as a modulator of Na_V_1.7 function across different cell types, thereby influencing their electrophysiological behavior.

### 4.2. Honokiol

Honokiol, a hydroxylated biphenyl compound isolated from *Magnolia officinalis* and other species within the Magnoliaceae family, has long been utilized in traditional Asian herbal medicines, including Houpo, Hou p’u, or Saiboku-to [77]. It is widely recognized as a promising natural compound with diverse biological activities, particularly due to its ability to influence multiple cellular processes across various cancer models [78]. In addition to its anticancer properties, previous studies have demonstrated that honokiol can regulate the functional activity of both neuroendocrine or endocrine systems. For instance, it has been shown to induce cell cycle arrest and trigger programmed cell death in human thyroid tumor cells, both in vitro and in vivo [79]. Furthermore, several studies have reported that *M. officinalis* bark and honokiol can modulate catecholamine secretion in adrenal chromaffin cells [80]. Beyond these effects, honokiol has also been found to exhibit antidepressant-like activity, potentially through normalization of the hypothalamic-pituitary-adrenal axis [81].

**Figure 7:**
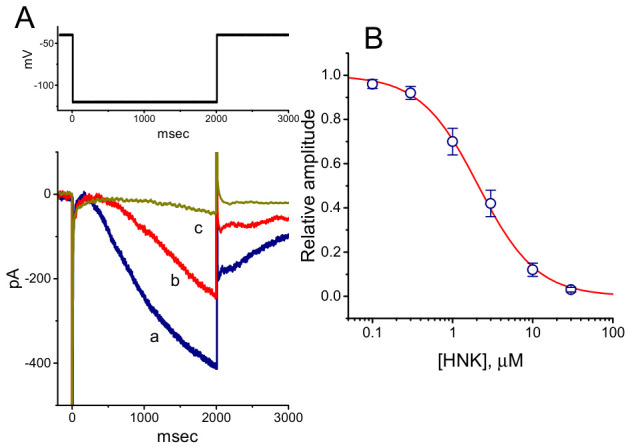
Concentration-dependent inhibition of the hyperpolarization-activated cation current (*I*_h_) by honokiol (HNK) in GH_3_ cells. (**A**) Representative *I*_h_ traces recorded under control conditions and following exposure to honokiol. Cells were perfused with Ca^2+^-free Tyrode’s solution containing 1 μM tetrodotoxin, and recording pipettes were filled with a K^+^-based internal solution. Hyperpolarizing voltage steps from −40 to −120 mV (2 s duration) were applied. Trace a corresponds to control conditions, while traces b and c were obtained after application of 3 μM and 10 μM honokiol, respectively. The upper part illustrates the voltage-clamp protocol used. (**B**) Concentration-response relationship for honokiol-mediated inhibition of *I*_h_ (mean ± SEM; n = 8 for each data point). The data were fitted with a red sigmoidal curve using the Hill equation, yielding an IC_50_ value of 2.1 μM. This figure is adapted from Ref. [82] and is distributed under the Creative Commons Attribution (CC BY) license.

Previous studies have shown that honokiol produces a concentration-dependent inhibition of *I*_h_ in GH_3_ cells, with an IC_50_ value of 2.1 μM (Figure 7) [82]. In addition to reducing current amplitude, honokiol shifts the steady-state activation curve of *I*_h_ toward a more negative potential without altering the apparent gating charge. This compound also attenuates the voltage-dependent hysteresis of *I*_h_ elicited by prolonged triangular ramp pulse. Moreover, honokiol has been reported to suppress *I*_h_ amplitude in Rolf B1.T olfactory neurons [82].

These findings collectively demonstrate that honokiol effectively inhibits *I*_h_ in both pituitary GH_3_ cells and Rolf B1.T olfactory neurons. The onset of this inhibition is rapid, suggesting that it may contribute to the compound’s modulatory effects on the functional activity of sensory neurons. Such suppression of *I*_h_ is likely to influence the firing behavior of electrically excitable cells, thereby altering neuronal excitability and over cellular function [82].

**Figure 8:**
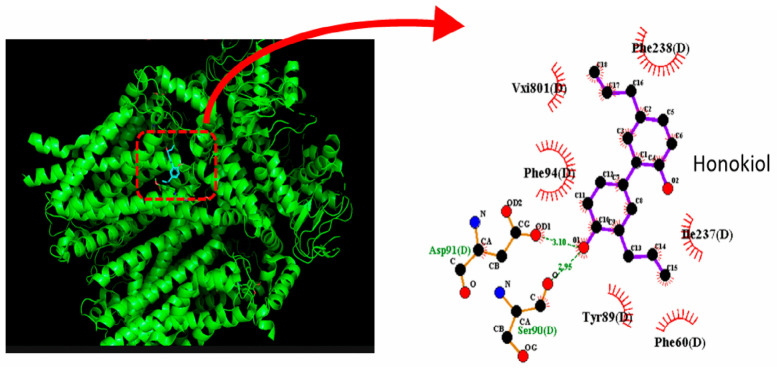
Predicted docking between the HCN3 channel and the honokiol molecule. The left panel depicts the HCN3-channel protein structure acquired from PDB (PDB ID: 8IO3), and the three-dimensional structure of honokiol from PubChem (Compound CID: 72303). PyRx software was used to show that the HCN3 channel structure can be optimally docked by the honokiol molecule. The right panel depicts an expansion of the red dashed box with a curved arrow in the left panel. This diagram of the interaction between the HCN3 channel and the honokiol was generated using LigPlot^+^ Version 2.3.

To examine how honokiol interacts with the HCN channel protein, we used the structure of HCN3 protein from PDB. The HCN3 channel protein structure was acquired from PDB (PDB ID: 8IO3), and PyRx 0.8 software was used to see how the honokiol molecule docks into the channel protein. Figure 8 presents the predicted docking sites of honokiol for interaction with amino-acid residues of the HCN3 channel. Specifically, the honokiol molecule was predicted to form hydrophobic contacts with residuals Phe60(D), Tyr89(D), Phe94(D), Ile237(D), and Phe238(D), as well as hydrogen bonds with residuals Ser90(D) and Asp91(D), with estimated distances of 2.95 and 3.10 Å, respectively. It is therefore anticipated that honokiol can interact with HCN channels to modulate the amplitude and gating kinetics of *I*_h_ [82].

**Figure 9:**
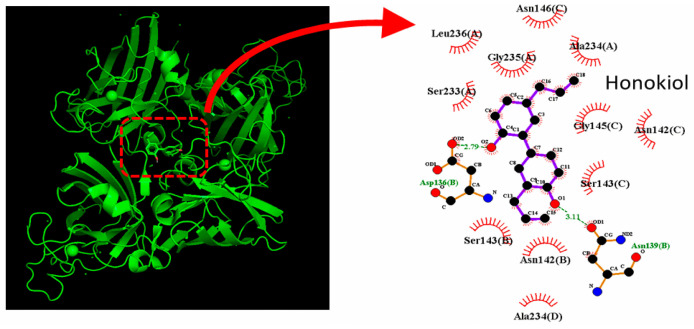
Molecular docking analysis of honokiol with superoxide dismutase. The protein structure of superoxide dismutase was acquired from PDB (PDB: 1DO5). The structure of superoxidase dismutase was docked with the honokiol molecule by using PyRx software, as indicated in the left side. The diagram of the interaction between superoxide peroxide protein and the honokiol molecule was generated by LigPlot+ (right side). The small green dots represent water molecules within the structure, with a total of 26 atoms included in this group.

Recent reports have demonstrated the ability of honokiol to exert antioxidant, anti-inflammatory, neurotrophic, and anti-apoptotic effects [83]. We also conducted a predicted docking analysis between honokiol and superoxide dismutase (SOD). SOD is a key antioxidant enzyme that plays a central role in controlling oxidative stress and modulating inflammation. We explored how SOD protein could be optimally docked with honokiol molecule by using PyRx software. The protein structure of SOD was obtained PDB (PDB ID: 1DO5). The predicted docking sites of the honokiol molecule with which the amino-acid residues can interact are presented in Figure 9. Notably, the honokiol molecule was predicted to form hydrophobic contacts with certain residues, including Asn142(B), Asn142(C), Ser143(B), Ser143(C), Gly145(C), Asn146(C), Ser233(A), Ala234(A), Gly235(A), and Leu236(A). The honokiol molecule was also predicted to form a hydrogen bond with residue Asp136(B) with a distance of 2.79 Å. These docking results suggest that honokiol can bind to SOD protein with a binding affinity of −7.4 kcal/mol. In high-resolution X-ray diffraction structures, the remaining water molecules are those associated with the protein, where other PDB structures, such as 8I5M (KCN10 channel), 6N4Q (SCN9A channel), and 8IO3 (HCN3 channel), determined by electron microscopy, do not display any water atoms.

### 4.3. Ganoderma Triterpenoids

*Ganoderma* mushrooms (known as Língzhī in Chinese and Reishi in Japanese) have long been used in traditional Chinese medicine, and are now recognized worldwide as a dietary supplement. Among the various species, *Ganoderma lucidum* (family Ganodermataceae) is the most extensively utilized and is commonly cultivated under controlled conditions to ensure a consistent chemical profile [84].

*G. lucidum* has been reported to exhibit a broad range of pharmacological activities, including antihypertensive, hypoglycemic, and hypocholesterolemic effects, along with other medicinal benefits [84,85]. Its major bioactive constituents are primarily triterpenoids [85,86]. In addition, *G. lucitum* has been shown to confer cardioprotective effects in animal models, likely by reducing oxidative stress associated with myocardial injury [87].

Triterpenes are a class of terpenes characterized by a C_30_ carbon backbone. Triterpenoids, their oxidized derivatives, typically have molecular weights ranging from 400 to 600 Da and exhibit structurally complex and highly oxidized chemical frameworks [88]. The triterpenoid fraction of *Ganoderma*, which includes more than 300 lanostane-type compounds, has been increasingly recognized for its biological activities. These include potent antioxidant effects the contribute to the prevention of myocardial injury, as well as notable neuroprotective properties [84,86,87,88].

In addition, *Ganoderma* triterpenoids have been shown to suppress inflammatory response by directly scavenging free radicals and by enhancing endogenous antioxidant enzymes, thereby reducing lipid peroxidation in animal models such as chicken liver and mice [87,89]. Furthermore, aqueous extract of *Ganoderma* has been reported to exhibit anticonvulsant, antidepressive, anxiolytic, and antinociceptive effects [90].

Previous studies have shown that the inhibitory effects of *Ganoderma* triterpenoids of *I*_h_ in GH_3_ cells extend beyond a simple reduction in current magnitude. These compounds also modify the kinetic properties of *I*_h_, indicating a dose-, time- and state-dependent mode of inhibition [91]. In addition, the presence of *Ganoderma* triterpenoids shifts the steady-state activation curve of *I*_h_ along the voltage axis toward more hyperpolarized potentials.

The IC_50_ value for *Ganoderma* triterpenoids-mediated inhibition of *I*_h_ observed in GH_3_ cells (11.7 μg/mL) is comparable to the concentrations previously reported to produce antioxidative and neuroprotective effects [85,91]. These findings suggest that the modulation of *I*_h_ may represent an early, upstream mechanism contributing to the compounds’ overall pharmacological actions, including their ability to regulate intracellular oxidative stress [84,85].

### 4.4. Sesamin and Sesamolin

Sesame seeds and sesame oil, derived from *Sesamum indicum* (family Pedaliaceae), have long been valued as health-promoting foods in many Asian countries [83]. Compared with edible oils obtained from other plant seeds, sesame oil exhibits remarkably oxidative stability. This stability is largely attributed to its high content of lipid-soluble furofuran lignans, particularly sesamin and sesamolin, which possess potent antioxidant properties [92].

Recent studies have highlighted that sesamin and sesamolin, the principal furofuran lignans found in sesame oil, possess a wide range of biological activities. These compounds have been shown to inhibit lipid peroxidation in erythrocytes [93], reduce intestinal cholesterol absorption, and suppress hepatic 3-hydroxy-3-methylglutaryl coenzyme-A (HMG-CoA) reductase activity [94]. In addition, they have demonstrated chemopreventive effects against chemically induced mammary carcinogenesis, as well as the ability to inhibit Δ5-desaturase activity and the elongation of C18 fatty acids. Both lignans also provide protective effects in hypoxic neuronal and PC12 cells, primarily through the suppression of reactive oxygen species (ROS) generation and mitogen-activated protein kinase (MAPK) signaling pathways [95]. Furthermore, sesamin and sesamolin have been reported to exert antihypertensive and cardioprotective effects [96].

Previous investigations have demonstrated that sesamin and sesamolin exert differential and potent inhibitory effects on both the transient and late components of *I*_Na_ in pituitary GH_3_ cells, in a concentration-dependent manner [97]. Specifically, sesamin was found to reduce the peak and sustained *I*_Na_ with IC_50_ values of 7.2 and 0.6 μM, respectively [97]. In addition to decreasing current amplitude, sesamin alters the inactivation kinetics of *I*_Na_ during brief depolarizing pulses.

Based on a Markovian model derived from the SCN8A channel [97,98], the sesamin-induced modulation of Na_V_ channel activity is thought to result from a decreased probability of channel occupying the open (O) and open-blocked (OB) states. These findings indicate that inhibition of Na_V_ channels by sesamin may represent a key ionic mechanism underlying its ability to modulate the functional behavior of various electrically excitable cells, provided that similar effects occur in vivo [98]. Furthermore, sesamin and structurally related lignans may act as modulators of Na_V_ channels in neoplastic or neuroendocrine tumor cells, suggesting potential applications in cancer neuroscience through the suppression of tumor progression, invasiveness, and metastasis [76,99].

### 4.5. Squamocin

Squamocin is a bis-tetrahydrofuran acetogenin that has been isolated from various members of Annonaceae family, including *Annona squamosa* (custard apple), *Annona muricata* (soursop), and *Asimina triloba* (pawpaw). Structurally, it features an extended aliphatic chain terminated by an α,β-unsaturated γ-lactone moiety, along with two tetrahydrofuran rings and several oxygen-containing functional groups distributed along the backbone.

This compound has attracted attention due to its diverse biological activities. It exhibits notable insecticidal properties [100] and has demonstrated potential anti-cancer effects [101]. Mechanistically, squamocin has been reported to inhibit mitochondrial NADH:ubiquinone oxidoreductase (complex I) [102], thereby affecting cellular energy metabolism. Furthermore, studies on asimicin, a structurally related analogue, suggest that its cytotoxic effects may involve alterations in membrane structure and dynamics [103]. More recent findings indicate that squamocin can trigger apoptosis in HL-60 leukemia cells, an effect that appears to be mediated through the activation of stress-activated protein kinase [104].

Earlier investigations have reported that squamocin exerts a stimulatory effect on Ca^2+^-activated K^+^ current (*I*_K(Ca)_) in human coronary smooth muscle cells [105]. This enhancement appears to be closely associated with elevations in intracellular Ca^2+^ levels. Supporting this interpretation, the observed increase in *I*_K([Ca2+]i)_ amplitude induced by squamocin is markedly reduced when extracellular Ca^2+^ is removed, indicating that both Ca^2+^ influx from the extracellular space and Ca^2+^ release from intracellular stores contribute to the overall response. These findings suggest that squamocin may facilitate the opening of membrane ion channels with high Ca^2+^ permeability [105], thereby elevating intracellular Ca^2+^ and secondarily augmenting *I*_K(Ca)_. Nevertheless, the precise cellular and molecular mechanisms underlying the actions of squamocin and related acetogenins across different cell types remain incompletely understood and warrant further investigation.

## 5. Polyphenols

### 5.1. Curcumin

Curcumin (diferuloylmethane, 1*E*,6*E*-1,7-bis(4-hydroxy-3-methoxyphenyl)-1,6-heptadiene-3,5-dione), a major constituent of the spice turmeric obtained from *Curcuma longa* (Zingiberaceae), is a bright yellow diarylheptanoid produced by some plants. It is the principal curcuminoid of turmeric (*Curcuma longa*), a member of the ginger family (Zingiberaceae). This compound is largely used as a herbal supplement, cosmetics ingredient, food flavoring and food coloring [106]. Particularly, this nutraceutical compound has been demonstrated to possess beneficial properties in a variety of diseases ranging from cancer to diabetes mellitus [107]. For example, curcumin has been recently reported to influence insulin release from isolated pancreatic islets [108].

Curcumin has been reported to exert a concentration- and state-dependent depressant action on *I*_K(DR)_ in pancreatic β-cells, particularly in the rat insulinoma cell line INS-1 [109]. The inhibitory action on this K^+^ current tends to correlate in time with a significant increase in the inactivation rate of the currents in response to membrane depolarization, while the activation kinetics of the current remained unaltered in the presence of curcumin [109].

The estimated peak of plasma curcumin concentration was previously reported to reach 3.14 μg/mL (around 8.57 μM) [110], a value which is apparently greater than the *K*_D_ (1.26 μM) and IC_50_ (3.32 μM) required for curcumin-mediated inhibition of *I*_K(DR)_ seen in INS-1 cells [109]. Therefore, the inhibitory effects of curcumin and curcuminoids on these K^+^ currents in pancreatic β-cells are expected to occur at concentrations achievable in the human organisms, although whether the effects of curcumin and other curcuminoid derivatives on ionic currents in humans still remains to be further delineated.

### 5.2. Columbianadin

Columbianadin is a prominent bioactive compound isolated from the root of *Angelica pubescens* Maxim. f. *biserrata* (Apiaceae), a medicinal plant commonly known as *Angelicae Pubescentis Radix*, or “Duho (Dú huó)” in traditional Chinese medicine. Chemically, columbianandin belongs to the coumarin family and is classified as an angular dihydrofuranocoumarin.

This compound has been widely recognized for its diverse pharmacological properties, including analgesic, anti-inflammatory, and anti-neoplastic activities [103,104]. It has been shown to influence cellular processes by modulating proliferation and promoting apoptosis [111]. In addition, experimental studies have demonstrated that columbianadin can attenuate inflammatory responses triggered by agents such as carrageenan and lipopolysaccharide, further supporting its potential therapeutic value in inflammatory conditions [111,112].

It is noteworthy that columbianadin suppresses *I*_Na_ in GH_3_ cells in a manner that depends on concentration and channel state [113]. Detailed analyses indicate that it exerts a differential inhibitory effect on the transient (peak) and sustained (late) components of *I*_Na_ elicited by rapid membrane depolarization with respective IC_50_ values of 14.7 and 2.8 μM [113]. In addition, columbianadin shifts the midpoint of the steady0-state inactivation curve toward more negative potentials, while leaving the activation time course of the current largely unaffected.

Further observations show that columbianadin can counteract the tefluthrin-induced enhancement of persistent *I*_Na_ during both the ascending and descending phases of an upright isosceles-triangular ramp. Tefluthrin, a pyrethroid insecticide known to augment *I*_Na_ [114,115], thereby serves as a useful pharmacological tool in this context.

In HL-1 cardiomyocytes, columbianadin similarly reduces *I*_Na_ amplitude and accelerates the slow component of current inactivation [113]. Collectively, these findings suggest that the compound modulates both the magnitude and gating kinetics of *I*_Na_. Such electrophysiological effects are likely to occur upstream of its reported influence on intracellular signaling pathways, including cytosolic NOD1/NF-κB activation [116] and on the regulation of antioxidant enzyme activity [117]. Consequently, these ion channel-modulating properties may contribute to the broader physiological actions of columbianadin in electrically excitable cells, such as GH_3_ cells or HL-1 cardiomyocytes, under in vivo conditions.

### 5.3. Eugenol

Eugenol (4-allyl-2-methoxyphenol) is an aromatic molecule found in several plants including clove (*Syzygium aromaticum*, Myrtaceae), bay leaves (*Laurus nobilis*, Lauraceae), and allspice (*Pimenta dioica*, Myrtaceae), and has been used in dental practice to relieve pain arising from a variety of sources, such as pulpal inflammations and dentin hypersensitivity [118]. Furthermore, eugenol is neuroprotective against excitotoxicity, cerebral ischemia and the toxic effects of amyloid-β peptides [119,120]. This compound has been demonstrated to suppress epileptiform field potentials and spreading depression in hippocampus and neocortex. These results suggest that the anti-convulsive properties of eugenol are mediated through the effects on neuronal ion fluxes by blocking Na_V_ channels [121,122,123].

The evidence has emerged that the effects on ion channels may be an important mechanism underlying eugenol-induced actions in neurons. Previous studies demonstrated the ability of eugenol to bind to vanilloid receptors and, in turn, to activate non-selective cation channels [124]. A recent work has also reported that this compound produced an inhibition of voltage-gated Ca^2+^ current through Ca_V_2.3 channels in the E52 cell line [124]. Such inhibition is thought to be direct and independent of the binding to vanilloid receptors or to β-adrenergic receptors [124,125].

Previous reports have demonstrated that the exposure to eugenol differentially inhibited the transient and late components of *I*_Na_ in differentiated NG108-15 neuronal cells in a concentration-dependent manner [126]. The IC_50_ values of eugenol required for the inhibition of the transient and late *I*_Na_ were 8.9 and 1.6 μM, respectively [126]. Eugenol can also diminish the amplitude of persistent *I*_Na_ evoked by long-lasting ramp pulse, and tefluthrin reversed the eugenol-induced inhibition of this current. This compound could decrease the frequency of spontaneous action potentials. It is therefore anticipated that the observed effects of this agent on ionic currents could be an important mechanism underlying its actions in neurons occurring in vivo [120,123,126].

### 5.4. Resveratrol

Resveratrol (trans-3,4′,5-trihydroxystilbene) is a polyphenolic phytoalexin naturally occurring in a variety of plant sources, such as grape (*Vitis vinifera*, family Vitaceae), peanuts (*Arachis hypogaea*, family Fabaceae), and several types of berries. A growing body of evidence has demonstrated that resveratrol exerts a wide range of pharmacological actions, including anti-platelet, anti-carcinogenic, anti-viral, and cardioprotective effects [127]. In addition, increasing studies suggest that resveratrol possesses notable neuroprotective properties [3,127,128,129]. Specifically, it has been shown to mitigate convulsions and neurotoxicity induced by kainic acid, as well as to confer protection against seizures triggered by pentylenetetrazol [130,131].

A notable observation is that resveratrol suppresses action potential firing in rat cortical neurons, and effect that appears to involve both enhancement of *I*_K(Ca)_ and inhibition of *I*_Na_ [132]. The increase in *I*_K(Ca)_ amplitude is thought to result from activation of large-conductance Ca^2+^-activated K^+^ channels [126,132,133]. In parallel, the inhibitory action of resveratrol on *I*_Na_ has been proposed to contribute to its analgesic properties. Through these concurrent modulatory effects on *I*_K(Ca)_ and *I*_Na_, resveratrol may hold promise as a broad-spectrum anti-seizure agent.

### 5.5. Gastrodigenin and Gastrodin

Gastrodigenin (*p*-hydroxybenzyl alcohol, also known as 4-hydroxybenzyl alcohol) is a phenolic constituent derived from the traditional Chinese medicinal herb *Gastrodia elata* Blume (family Orchidaceae), commonly referred to as Tian Ma in Chinese and Chunma in Korean [134]. Its glucoside form, gastrodin, represents the principal bioactive compound isolated from this plant. For centuries, the dried rhizomes of *G. elata* have been extensively utilized for the management of various neurological and psychiatric conditions, including seizures, vertigo, cognitive decline, depressive disorders, and migraines [135,136,137,138]. In recent years, growing attention has been directed toward elucidating the molecular and cellular mechanisms by which the active components of *G. elata* produce their diverse pharmacological effects.

Previous investigations have examined the effects of two structurally related compounds, gastrodigenin and its glucoside gastrodin, on various ionic currents in pituitary cells and hippocampal neurons. In pituitary GH_3_ lactotrophs, both agents were found to suppress the amplitude of *I*_K(M)_ in a time- and concentration-dependent manner [131]. The IC_50_ value for gastrodigenin and gastrodin in these cells were estimated to be 12.1 and 19.4 μM, respectively [138].

Notably, the inhibition of *I*_K(M)_ by gastrodigenin was accompanied by a slowing of the current’s activation kinetics, whereas gastrodin produced an apparent acceleration of the activation time course. In addition, gastrodigenin shifted the steady-state activation curve of *I*_K(M)_ toward more hyperpolarized potentials without altering the apparent gating charge [138]. Both components were also effective in reducing *I*_K(M)_ amplitude in hippocampal mHippoE-14 neurons.

Given that gastrodin is capable of crossing the blood–brain barrier, these findings suggest that modulation of ionic currents—particularly low threshold *I*_K(M)_, which is characterized by slow activation and deactivation kinetics—may represent an important mechanism underlying the actions of these compounds in endocrine or neuroendocrine cells, as well as in central neurons, provided that similar effects occur in vivo [136,137,138].

### 5.6. Pterostilbene

Pterostilbene (trans-3,5-dimethoxy-4′-hydroxystilbene) is a natural dimethylated analog of resveratrol isolated from *Pterocarpus marsupium* Roxb. (Fabaceae), a tree native to India, Nepal, and Sri Lanka. The extracts of *P. marsupium* containing pterostilbene have been traditionally used in Ayurvedic medicine for treating various disorders [139]. This compound has been reported to have benefits for the prevention or treatment of different kinds of cancers, as mounting evidence has demonstrated its inhibitory effects on almost every cellular event that promotes tumor progression toward metastasis in both apoptosis-dependent and apoptosis-independent manners [140,141,142].

**Figure 10:**
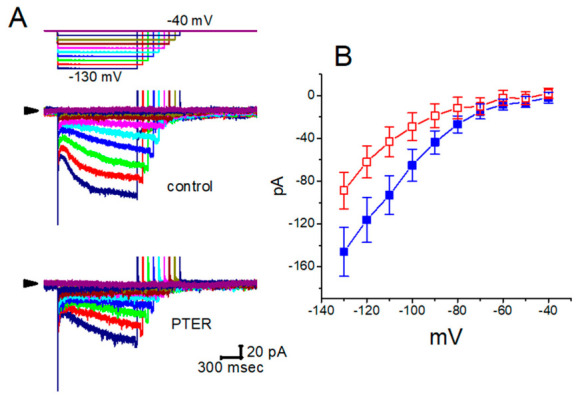
Effect of pterostilbene (PTER) on the current-voltage (I–V) relationships of *I*_h_ in GH_3_ cells. Current traces were recorded during 2-s voltage steps to a family of membrane potentials ranging between −130 and −40 mV in 10-mV steps from a holding potential of −40 mV, as indicated in the uppermost part of (**A**). (**A**) Representative *I*_h_ traces achieved under the control condition (i.e., pterostilbene was not present) (upper) and during the exposure to 1 μM pterostilbene (lower). Arrowhead in each panel depicts the zero-current level and the calibration mark shown in the right lower side applies to all current traces. Of note, there are varying durations in voltage-clamp profile for better illustration. (**B**) Averaged I–V relations of *I*_h_ achieved in the absence (■) and presence (□) of 1 μM pterostilbene (mean ± SEM; n = 9 for each data point). Current amplitude was measured at the end of each voltage step. This figure is adapted from Ref. [143] and is published under the Creative Commons Attribution (CC BY) license.

In earlier studies, the addition of pterostilbene inhibited *I*_h_ effectively in a concentration- and time-dependent manner in pituitary GH_3_ cells. The I–V relationships of *I*_h_ established at various levels of hyperpolarizing steps were also established and depicted in Figure 10. It was noted that the presence of pterostilbene (1 μM) significantly reduced the slope of the linear fit of *I*_h_ amplitudes to the voltages between −130 and −100 mV from 27.2 ± 1.3 to 13.1 ± 1.1 nS (n = 8, *p* < 0.05). The steady state activation curve of *I*_h_ was distinctly shifted to more hyperpolarizing potentials by 11 mV, producing channel opening at more negative voltages. The results thus demonstrate that pterostilbene has a conceivable depressant action on *I*_h_ functionally expressed in GH_3_ cells [143].

**Figure 11:**
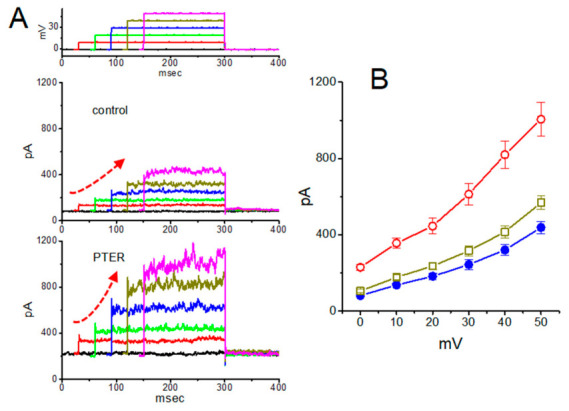
Stimulatory effect of pterostilbene (PTER) on Ca^2+^-activated K^+^ current (*I*_K(Ca)_) identified in GH_3_ cells. This set of whole-cell current recordings was conducted in cells suspended in normal Tyrode’s solution which contained 1.8 mM CaCl_2_. (**A**) Representative *I*_K(Ca)_ traces obtained during the voltage steps to various membrane potentials between 0 and +50 mV in 10-mV steps from a holding potential of 0 mV (as indicated in the uppermost part of (**A**)). Records shown in the upper and lower panels were obtained under control condition and during the exposure to 3 μM pterostilbene, respectively. The uppermost part depicts the voltage pulses applied. The red dashed curved arrow indicates that the amplitude of *I*_K(Ca)_ increases as the cell membrane depolarizes, demonstrating its outwardly rectifying properties. (**B**) Averaged I–V relations of *I*_K(Ca)_ obtained in the control (**●**), during the exposure to 3 μM pterostilbene (**○**), and after washout of the agent (**□**) (mean ± SEM; n = 8 for each data point). This figure is adapted from Ref. [143] and is published under the Creative Commons Attribution (CC BY) license.

Furthermore, as shown in Figure 11, the presence of pterostilbene raised the amplitude of macroscopic *I*_K(Ca)_ in GH_3_ cells. In this set of whole-cell current recordings, GH_3_ cells were bathed in normal Tyrode’s solution containing 1.8 mM CaCl_2_, and the recording pipette was filled with a K^+^-enriched solution. In an attempt to inactivate most of the voltage-gated K^+^ currents, we then maintained the examined cells at the level of 0 mV, and then applied a series of voltage pulses between 0 and +50 mV with 10-mV steps. Within 1 min of exposing GH_3_ cells to pterostilbene (3 μM), the amplitude of *I*_K(Ca)_ elicited by this voltage profile evidently rose (Figure 11). For example, the addition of 3 μM pterostilbene increased *I*_K(Ca)_ amplitude elicited by depolarizing pulse from 0 to +50 mV from 568 ± 35 to 1005 ± 89 pA (n = 8, *p* < 0.05). As the compound was washed out, current amplitude returned to 621 ± 38 pA (n = 8). The averaged I–V relationships of *I*_K(Ca)_ amplitude in the control, during the exposure to 3 μM pterostilbene and after washout of the compound were depicted in Figure 11B. The addition of 3 μM pterostilbene substantially increased the whole-cell conductance of *I*_K(Ca)_ measured at the voltages between +30 and +50 mV to 19.6 ± 0.8 nS (n = 8, *p* < 0.05) from control value of 12.6 ± 0.5 nS (n = 8). Therefore, pterostilbene can increase the amplitude of *I*_K(Ca)_ observed in these cells and its stimulation of *I*_K(Ca)_ was largely attributable to the activation of large-conductance Ca^2+^-activated K^+^ channels [143].

## 6. Other Aromatic or Conjugated Systems

### 6.1. Isoplumbagin and Plumbagin

Isoplumbagin (5-hydroxy-3-methyl-1,4-naphthoquinone) is a naturally occurring quinone from *Lawsonia inermis* (Lythraceae) or *Plumbago europarea* (Plumbaginaceae) [144]. Similar to isoplumbagin, plumbagin (5-hydroxy-2-methyl-1,4-naphthoquinone), another hydroxyl-1,4-napththoquinone, is another alkaloid obtained from the roots of the plants of *Plumbago* genus. Isoplumbagin and plumbagin have been demonstrated to exert antineoplastic activity against an array of cancer cells, including oral and tongue squamous cell carcinoma, glioblastoma, non-small cell lung carcinoma, and breast, cervical, endometrial, pancreatic and prostate cancers [145,146]. Alternatively, plumbagin was shown to induce apoptotic changes in lung cancer and neuronal cells via caspase-9 activation and targeting mitochondrial-mediated ROS induction [147,148]. Plumbagin has been reported to modify release of pituitary gonadotropin [149]. Previous studies have shown the ability of 2-mercaptophenyl-1,4-naphthoquinone, a naphthoquinone derivative, to overcome the elevation of intracellular Ca^2+^ in platelets caused by ADP and collagen [150].

Accumulating evidence indicates that isoplumbagin can influence the magnitude, gating behavior, and voltage-dependent hysteresis of *ether-à-go-go*-related gene (*erg*)-mediated K^+^ currents (*I*_K(erg)_) recorded in GH_3_ cells [151]. Moreover, these *erg*-mediated K^+^ currents were similarly suppressed by isoplumbagin in MA-10 Leydig tumor cells [151]. These findings suggest that the inhibitory effects of isoplumbagin, as well as the structurally related compound plumbagin, on *I*_K(erg)_ may play a role in their anti-neoplastic properties, provided that such effects are recapitulated under in vivo conditions.

The IC_50_ values for isoplumbagin-mediated suppression of the peak and sustained components of *I*_K(erg)_ were determined to be 18.3 or 2.4 μM, respectively [151]. Based on a first-order reaction scheme, the dissociation constant (K_D_) was estimated to be 2.58 μM, a value closely consistent with the IC_50_ (i.e., 2.4 μM) for inhibition of the sustained *I*_K(erg)_ elicited by prolonged membrane hyperpolarization.

Previous work has also demonstrated that isoplumbagin, at a concentration of 2.5 μM, can impair mitochondrial respiration, likely through inhibition of complex IV activity [152]. In addition, the extent to which isoplumbagin or plumbagin modulates *I*_K(erg)_ is influenced not only by their concentration but also by several contextual factors, including the baseline resting membrane potential and patterns of action potential firing, assuming that *I*_K(erg)_ is sufficiently expressed in the cells under study.

Although the precise mechanisms underlying isoplumbagin-induced inhibition of *I*_K(erg)_ remains to be fully elucidated, such blockade is likely to be of pharmacological significance [8]. Notably, it has been proposed that isoplumbagin or plumbagin may directly enhance prolactin secretion in vivo via suppression of *I*_K(erg)_ [8,153]. However, the potential impact of isoplumbagin-mediated *I*_K(erg)_ inhibition on cardiac function [153,154] has yet to be clearly defined.

### 6.2. Verteporfin

Verteporfin (Visudyne^®^), a benzoporphyrin derivative, is a clinically utilized photosensitizer in photodynamic therapy. It is specifically designed to selectively ablate abnormal ocular vasculature, such as that associated with neovascular (wet) age-related macular degeneration and choroidal neovascularization [155,156,157].

Photodynamic therapy employing photosensitizers, including hypericin and verteporfin, has also been shown to be effective in treating a variety of hyperplastic and neoplastic conditions, such as residual tumors in the pituitary gland [158,159]. In line with this, earlier studies demonstrated that certain photosensitizers—for example, rose Bengal, a fluorescein-derived compound—can modulate membrane ionic current in GH_3_ cells as well as in cardiac cells [160]. Conversely, there is evidence indicating that verteporfin may produce adverse effects; specifically, it has been reported to induce anterior ischemic optic neuropathy in rodent models [155,161].

A prior study reported that verteporfin exerts a pronounced stimulatory effect on *I*_K(Ca)_ in pituitary GH_3_ cells, while producing only a modest reduction in the amplitude of *I*_K(DR)_, without significantly affecting either *I*_Na_ or *I*_K(M)_ [5]. The enhancement of *I*_K(Ca)_ by verteporfin is thought to arise from alterations in intracellular Ca^2+^ levels. Supporting this notion, the verteporfin-induced increase in *I*_K(Ca)_ was markedly attenuated when extracellular Ca^2+^ was removed, indicating that both Ca^2+^ influx from the extracellular space and release from intracellular stores contribute to the observed augmentation of *I*_K(Ca)_ in these cells.

The EC_50_ value for either verteporfin-induced enhancement of *I*_K(Ca)_ in pituitary GH_3_ cells and for the increased activity of large-conductance Ca^2+^-activated K^+^ channels in 13-06-MG glioma cells (a human glioblastoma multiforme cell line) was estimated to be 2.4 or 1.9 μM, respectively [5]. Notably, these concentrations are comparable to those that are either clinically attainable or required to inhibit the YAP-TEAD (Yes-associated protein-TEA domain transcription factor) complex in various anaplastic or neoplastic cells [6,162,163,164]. Given that verteporfin augments *I*_K(Ca)_ amplitude within a short time frame in GH_3_ cells, it is reasonable to propose a potential mechanistic link between its anti-neoplastic properties and its stimulatory effects on *I*_K(Ca)_. Such actions may occur rapidly, possibly even prior to significant intracellular accumulation of the compound [1,6,162].

## 7. Conclusions

This review illustrates that five major chemical classes of phytoconstituents exert distinct and specific modulatory effects on membrane ionic currents, providing a mechanistic basis for their potential pharmacological activities. Alkaloids predominantly exert inhibitory effects on voltage-gated K^+^ currents; however, aconitine specifically affects the inactivation of *I*_Na_ and *I*_K(DR)_. Terpenoids modulate *I*_K(M)_, *I*_h_, and *I*_K(DR)_. Compounds within the lignan and acetogenin groups exhibit inhibitory actions on *I*_Na_ and *I*_h_. Polyphenolic compounds influence, to varying extents, *I*_K(DR)_, *I*_Na_, *I*_h_, *I*_K(erg)_, *I*_K(M)_, and *I*_K(Ca)_. In contrast, other aromatic or conjugated compounds can regulate *I*_K(erg)_ and *I*_K(Ca)_. Moreover, individual phytoconstituents also display distinct specificities with respect to both the amplitude and gating kinetics of different ionic currents across various cell types.

Ion channel gating kinetics refers to the dynamic rates governing transitions among the functional states of a channel—closed, open, and inactivated—and encompasses activation, inactivation, deactivation, and recovery kinetics [6,114]. By utilizing electrophysiological techniques—particularly voltage-clamp methods—and incorporating digital-to-analog conversion to generate a variety of voltage-clamp profiles, this approach remains the principal method currently used to investigate voltage-clamp ionic currents [6]. The docking predictions also indicate that several amino acid residues within many ion channel structures are capable of forming molecular interactions with various phytoconstituents, including hydrogen bonds and hydrophobic contacts. Collectively, these findings have important pharmacological and toxicological implications for the various bioactivities of individual phytoconstituents [1,2,3,4,165,166,167,168,169,170].

In addition, phytoconstituents often modulate ion channel proteins situated on the cell surface or interact with nearby membrane phospholipids. Because these actions occur at or within the plasma membrane, the compounds do not necessarily need to enter the cell. As a result, their effects typically appear rapidly and extremely low concentrations are not required for efficacy [39,165,166,167,168]. In contrast, when their targets reside within the cytoplasm—such as intracellular signaling pathways—or within the nucleus, where they influence transcriptional or epigenetic processes, the onset of action is generally slower. Access to these intracellular targets requires the compounds to cross the plasma membrane, a process largely determined by their lipid solubility (e.g., reflected by the partition coefficient). Consequently, although some bioactive phytoconstituents appear to demonstrate biological effects at relatively low concentrations in cell-free assays, such as Western blotting, immunofluorescent assay, and co-immunoprecipitation [2,3,23,29,62,83,104,140,171], substantially higher concentrations are often required to elicit measurable intracellular responses [40,165,168].

In this paper, the docking studies have been performed using PyRx. The docking analysis is intended to only a preliminary, qualitative indication of potential binding modes rather than definitive structural conclusions. The limitations of the approach, including uncertainties in scoring accuracy and the lack of explicit solvent (water) representation in the docking protocol. Additionally, in the absence of experimental validation, molecular docking results should be interpreted with caution.

It is also important to recognize that, in addition to the phytoconstituents described in this article, numerous other compounds with significant modulatory effects on ion channels are likely to be discovered as electrophysiological methodologies—such as automated patch-clamp recording—continue to advance and gain wider application. Moreover, with the exception of certain ligand-gated channels and transient receptor potential (TRP) channels, voltage-gated ion currents are typically activated either rapidly—within a few milliseconds (e.g., *I*_Na_)—or more slowly, over seconds (e.g., *I*_K(erg)_ and *I*_h_) [1,40,159]. Moreover, the magnitude, gating kinetics, and voltage-dependent hysteresis of voltage-gated ionic currents vary significantly across different cell types [39,50,153,166,167]. In contrast, antineoplastic or antioxidative effects generally require several hours to become apparent. Therefore, elucidating how rapid modulation of ion channels translates into slower antineoplastic, antioxidative, or immunomodulatory outcomes represents an important direction for future research [1,2,6,166]. Bridging this temporal gap remains an important objective, as it may reveal ion channel activity as an early trigger of subsequent intracellular responses, including those occurring in the mitochondria, cytoplasm, or nucleus) [1,10,165,168].

## Data Availability

The data are available upon reasonable request to the corresponding author.

## Abbreviations

The following abbreviations are used in this manuscript:

*Erg*	*ether-à-go-go*-related gene
HCN channel	Hyperpolarization-activated cyclic nucleotide-gated channel
IC_50_	Concentration required for half-maximal inhibition
*I*_h_	Hyperpolarization-activated cation current
*I*_K(Ca)_	Ca^2+^-activated K^+^ current
*I*_K(DR)_	Delayed-rectifier K^+^ current
*I*_K(erg)_	*erg*-mediated K^+^ current
*I*_K(M)_	M-type K^+^ current
*I*_Na_	Voltage-gated Na^+^ current
Na_V_ (SCN) channel	Voltage-gated Na^+^ channel
I–V relationship	Current versus voltage relationship
